# Reinduction of Hedgehog Inhibitors after Checkpoint Inhibition in Advanced Basal Cell Carcinoma: A Series of 12 Patients

**DOI:** 10.3390/cancers14215469

**Published:** 2022-11-07

**Authors:** Viola K. DeTemple, Jessica C. Hassel, Michael M. Sachse, Imke Grimmelmann, Ulrike Leiter, Christoffer Gebhardt, Julia Eckardt, Claudia Pföhler, Yenny Angela, Hanna Hübbe, Ralf Gutzmer

**Affiliations:** 1 Department for Dermatology, Johannes Wesling Medical Center Minden, Ruhr University Bochum, 32429 Minden, Germany; 2 Section Dermatooncology, Department of Dermatology, National Center for Tumor Diseases, University Hospital Heidelberg, 69120 Heidelberg, Germany; 3 Skin Cancer Center, Clinic for Dermatology, Allergology and Phlebology, Hospital Bremerhaven, 27574 Bremerhaven, Germany; 4 Skin Cancer Center Hannover, Clinic for Dermatology, Allergology and Venerology, Hannover Medical School, 30163 Hannover, Germany; 5 Skin Cancer Centre Department of Dermatology, University of Tuebingen, 72074 Tübingen, Germany; 6 Department for Dermatology and Venerology, University hospital Hamburg-Eppendorf (UKE), 20251 Hamburg, Germany; 7 Skin Cancer Centre, Department for Dermatology, Venerology and Allergology, Charité, University Hospital Berlin, 10117 Berlin, Germany; 8 Skin Cancer Centre, Department for Dermatology, Venerology and Allergology, Saarland University Medical Center and Saarland University Faculty of Medicine, 66421 Homburg, Germany

**Keywords:** advanced basal cell carcinoma, hedgehog inhibitor reinduction, sequential treatment, checkpoint inhibition

## Abstract

**Simple Summary:**

For patients with advanced basal cell carcinoma (aBCC) limited treatment options are available. In this situation, hedgehog inhibitors (HHIs) are approved as first-line treatment. Upon treatment failure or intolerance, a second-line treatment with PD1 inhibitors (PD1i) is an option. However, no third-line treatment is established. Therefore, we collected data of patients with aBCC, who received HHI reinduction following PD1i-failure. In our cohort of 12 patients, initial HHI treatment led to partial response in 8 and disease stabilization in 4 patients. Eventual HHI discontinuation was mostly due to tumor progression. Second-line PD1i resulted in a partial response in only one patient. Four out of the twelve patients responded to HHI reinduction, with the longest follow-up period being 29 months. Thus, a sequential treatment with HHI reinduction can be a feasible treatment option in a subgroup of patients with aBCC after treatment failure of first-line HHIs as well as of second-line PD1i.

**Abstract:**

For patients with advanced basal cell carcinoma (aBCC) first-line treatment with hedgehog inhibitors (HHIs) and second-line treatment with PD1 inhibitors (PD1i) is available, offering combination and sequencing options. Here, we focus on the efficacy and safety of HHI reinduction after PD1i failure. Retrospective data analysis was performed with 12 patients with aBCC (locally advanced (n = 8)/metastatic (n = 4)). These patients (male:female 6:6, median age 68 years) initially received HHIs, leading to complete/partial response (66%) or stable disease (33%). Median treatment duration was 20.8 (2–64.5) months until discontinuation due to progression (n = 8), adverse events (n = 3), or patient request (n = 1). Subsequent PD1 inhibition (pembrolizumab 42%, cemiplimab 58%) yielded a partial response (8%), stable disease (33%), or progression (59%). Median treatment duration was 4.1 (0.8–16.3) months until discontinuation due to progression (n = 9), adverse events (n = 1), patient request (n = 1), or missing drug approval (n = 1). HHI reinduction resulted in complete/partial response (33%), stable disease (50%), or progression (17%). Median treatment duration was 3.6 (1–29) months. Response duration in the four responding patients was 2–29+ months. Thus, a subgroup of patients with aBCC responded to reinduction of HHI following PD1i failure. Therefore, this sequential treatment represents a feasible treatment option.

## 1. Introduction

Advanced basal cell carcinoma (aBCC) is a rare condition of either locally advanced (laBCC) or metastatic (mBCC) disease not amenable to surgical excision or radiotherapy [1]. A first classification is the European Association of Dermato-Oncology (EADO) staging system for basal cell carcinoma (BCC), based on clinical pattern recognition concerning the difficulty in treatment (difficulty-to-treat score) [2,3].

Vismodegib and sonidegib are oral hedgehog inhibitors (HHIs), which have shown efficacy and safety in the treatment of aBCC. HHIs bind selectively to Smoothened (SMO), a key protein within the hedgehog pathway, which leads to activation of the pathway in the majority of BCCs. Response rates of HHIs for laBCC were reported as 47.6% for vismodegib during a follow-up period of 21 months and 60.6% for sonidegib during a follow-up period of 18 months. Both substances were associated with overall comparable adverse events (AEs) including muscle spasms, alopecia, dysgeusia, weight loss, and fatigue. Although the majority of AEs were rated as mild, they can impair quality of life and frequently result in dose interruption or drug discontinuation [4].

Therapy with programmed cell death protein-1 inhibitors (PD1i) provide another option for patients with aBCC. The PD1i cemiplimab has been approved since 2021 for patients with aBCC intolerant to or progressive on treatment with HHIs. PD1i increase the T-cell-mediated immune response, thereby sustaining the endogenous antitumor activity [5,6]. It has been found that 31% of patients with laBCC and 24% with mBCC will respond to second-line treatment with cemiplimab after HHI. Even though PD1i pembrolizumab is at present not approved for treatment of aBCC, small studies and case series suggest a similar response rate in aBCC pretreated with HHI compared to cemiplimab [5,6,7,8].

These two therapeutic strategies, HHIs and PD1i, offer possibilities for sequencing and combination. In clinical practice, progression of aBCC following HHIs as well as subsequent PD1i treatment occurs, and no data on third-line treatment experience exist thus far. The aim of this study was to summarize data on efficacy and tolerability of HHI reinduction in this situation.

Therefore, we retrospectively collected data from German skin-cancer centers on patients with aBCC, who were reinduced with HHI after pretreatment with HHIs and PD1i.

## 2. Patients and Methods

German skin-cancer centers were requested to participate in this retrospective multicenter study. Patients qualified for the study if they fulfilled the inclusion criteria of aBCC treated with the sequence HHI–PD1i–HHI (between 2016 and 2022) and the retrospective availability of sufficient data to assess efficacy and tolerability of these treatments. Diagnosis of BCC was performed by local standards of care in the respective skin cancer centers. Eight skin-cancer centers (Berlin, Bremerhaven, Hamburg, Hannover, Heidelberg, Homburg, Minden and Tübingen) contributed 12 patients.

The following data were collected: age and disease stage at primary HHI induction; site of metastasis, if applicable; concomitant diseases; sex; type of HHI (vismodegib or sonidegib) at primary induction and reinduction; type of interjacent PD1i (pembrolizumab or cemiplimab); subsequent treatment for laBCC or mBCC, if applicable; duration of and best response to each treatment regime; adverse events; reasons for discontinuation; and clinical course from primary HHI induction until death or last observation.

Best response was assessed via clinical evaluation or radiographic imaging, as applicable, by each participating institution.

Toxicity was graded by each participating institution according to the National Cancer Institute Common Terminology Criteria for Adverse Events (CTCAE) V.5.0.

Calculations including medians and time intervals as well as percentual ratios were performed using Microsoft Excel (Microsoft Office 2019). This software was also used to generate the swimmers plot. Due to the small number of patients and the descriptive presentation of this clinically relevant scenario, no statistical tests were performed.

## 3. Results

In total, 12 aBCC patients who were treated with an HHI–PD1i–HHI therapy sequence between 2016 and 2022 were included in this study. At primary HHI induction, median age was 68 years (ranging from 31 to 87 years). Six patients were female (50%), six were male (50%). Eight patients (67%) presented with laBCC and four patients (33%) with mBCC at HHI induction (Table 1).

For primary HHI therapy, nine patients (75%) received vismodegib, while two patients (17%) received sonidegib. In one patient (8%, case 6) within the initial HHI cycle, therapy with vismodegib was switched to sonidegib due to disease progression. For interjacent PD1i therapy, five patients (42%) were treated with pembrolizumab and seven patients (58%) with cemiplimab. Reinduction of HHI therapy was performed with vismodegib in three patients (25%) and sonidegib in nine patients (75%). Four patients (33%) received the same HHI type at primary and reinduction treatment. One patient (8%) was treated with cetuximab and paclitaxel between PD1i and HHI reinduction (Table 2). Observation time ended with death for four patients (33%), of whom three died due to disease progression.

All patients had disease control during initial HHI treatment (complete response (CR) in one patient, partial response (PR) in seven patients, and stable disease (SD) in four patients). Treatment was discontinued due to disease progression in eight patients (67%), intolerable AEs in three patients (25%), and the patient’s own decision in one patient (8%). The median therapy duration was 20.8 months (range from 2 to 64.5 months).

The consecutive PD1i therapy achieved a low overall response rate. Seven patients experienced progressive disease (PD, 58%), four patients SD (33%), and one patient PR (8%). The median therapy duration was 4.1 months (range from 0.8 to 16.3 months). PD1i therapy was most frequently discontinued due to progression (nine patients, 75%). Other reasons were AEs (one patient, 8%), patient request (one patient, 8%), and lack of drug approval and reimbursement problems from treatment continuation (one patient, 8%).

**Figure 1:**
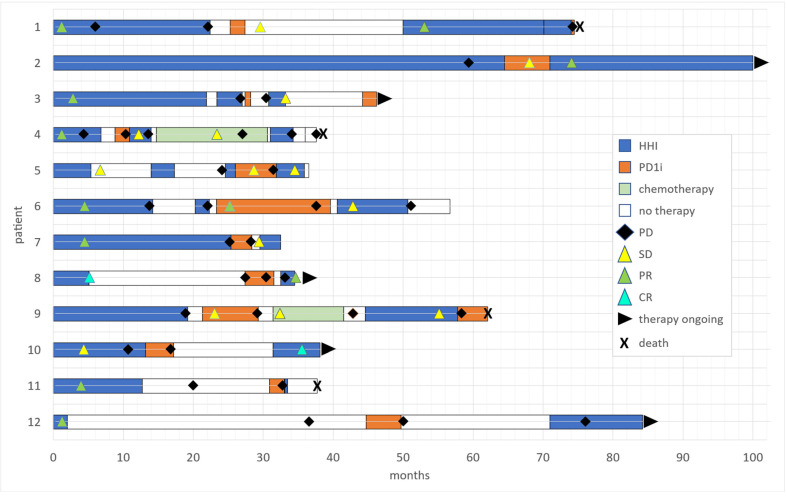
Course of treatment across all 12 patients depicted as swimmers plot.

**Figure 2:**
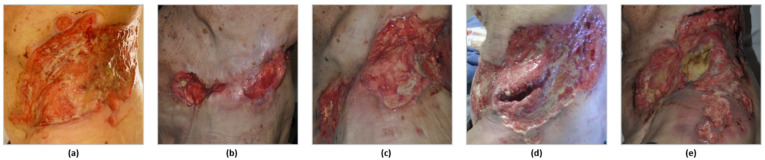
Exemplary photodocumentation of one patient during the course of treatment. Photodocumentation for patient 7 with locally advanced basal cell carcinoma along the treatment sequence HHI–PD1i–HHI. (**a**) Starting point before systemic treatment with locally advanced basal cell carcinoma on the left flank. (**b**) Initial partial response to vismodegib within 16 months of treatment, followed by (**c**) progressive disease under HHI after another 7 months. (**d**) Further progressive disease under cemiplimab within 6 months after only short term partial response. (**e**) Tumor stabilization was reached within 4 months under HHI reinduction with sonidegib.

HHI therapy was reinduced within a median of 18.3 months after discontinuation of initial HHI treatment (range from 3.8 to 69 months), and within a median of one month after discontinuation of PD1i treatment (range from 0 to 22.6 months). One third of patients had an objective response to the second HHI treatment, including three patients with PR (25%) and one patient with CR (8%). Six patients (50%) achieved a SD, two patients (16%) a PD. The median treatment duration was 3.6 months (range from 1 to 29 months). In total, seven patients had to discontinue the treatment with reinduced HHI, mostly due to progression (five patients, 42%), but also due to intolerable AEs (one patient, 8.3%) or patient request (one patient, 8.3%). Among these, five patients received subsequent systemic treatments. The remaining five patients are still on treatment with reinduced HHI. An overview of treatment sequences is summarized in Figure 1. Figure 2 depicts one exemplary clinical course (patient 7).

Overview of treatment course for the 12 patients in our cohort with locally advanced or metastasized basal cell carcinoma depicted as swimmers plot. Each bar represents one patient. Time in months is plotted on the *x*-axis. Color of the bar represents treatment regime. Marks within the bars depict best response (if time point was given). Ongoing treatment or death of patient is marked at the end of the bar, respectively.

In most patients, response to HHI reinduction was lower than response to initial HHI therapy (n = 8). Among them, one patient responded with PR to reinduced HHI after CR following initial HHI (primary discontinuation of HHI due to AEs). Five patients showed PR to initial HHI and only SD to HHI reinduction (primary discontinuation of HHI due to progression (n = 4) or patient request (n = 1)). Two patients (cases 11 and 12) showed no response to HHI reinduction despite PR or SD during initial HHI (primary discontinuation of HHI due to AEs).

Patients with comparable response to primary HHI and HHI reinduction presented with PR (case 1) and SD (case 5), respectively. In both cases, the reason for discontinuation of initial HHI was progression.

A further two patients presented with better response to reinduced HHI: one responded with PR to HHI reinduction (case 2) and the other with CR (case 10), both following SD after initial HHI. In both cases, the reason for discontinuation of primary HHI was progression.

All patients with initial PR as best response (n = 4) achieved a worse best response during HHI reinduction. Two patients discontinued the initial HHI treatment due to PD and one patient could not continue the treatment due to lack of drug approval and reimbursement. These three patients showed tumor stabilization upon secondary HHI treatment.

One patient with CR after initial HHI treatment discontinued the treatment due to AEs. Following HHI reinduction best response was documented as PR.

Thus, response to HHI reinduction appears to be independent from response to primary HHI treatment.

All patients with discontinuation of initial HHI treatment due to AEs or patient request (n = 4) were no longer responsive to HHI reinduction (SD, n = 2; PD, n = 2).

The remaining patients (n = 8), who had to discontinue primary HHI treatment due to tumor progression, presented with worse (n = 4), similar (n = 2), but also better (n = 2) response to HHI reinduction compared to initial HHI treatment. None of these patients had progressive disease upon HHI reinduction.

Here, one can detect a tendency, that discontinuation of primary HHI treatment due to progression may be associated with a higher probability of response to HHI reinduction.

Patients with mBCC (n = 4) reached PR (n = 3) or SD (n = 1) during initial HHI treatment. In all four cases, the reason for discontinuation was progression. Response to HHI reinduction was comparable in two cases (PR in case 1, SD in case 5). Two cases showed a lower response rate (both SD, cases 4 and 7). None of these patients showed progressive disease as objective response upon HHI reinduction.

Patients with laBCC (n = 8) showed a more diverse response pattern. Best response to initial HHI treatment ranged from SD (n = 3) to PR (n = 4) to CR (n = 1). The reason for discontinuation was progression (n = 4), AEs (n = 3), or patient request (n = 1).

For laBCC patients with initial SD as best response (n = 3) upon the first cycle of HHI and discontinuation due to PD (n = 2), two patients experienced a better response (PR, n = 1; CR, n = 1) during HHI reinduction, while one patient, who had to be discontinued due to AEs, was no longer responsive to secondary HHI treatment.

Hence, stage of aBCC does not seem to correlate with response to HHI reinduction.

In our patient group, observed AEs corresponded with those previously reported. During initial HHI nearly all patients experienced AEs (n = 11), leading to discontinuation in three patients. AEs under HHI reinduction occurred with a lower frequency (n = 6). Some AEs reoccurred. Overall, PD1i was better tolerated than HHIs with only three patients suffering AEs. Notably, PD1i treatment as well as HHI reinduction periods were shorter than the initial HHI treatment episodes (Table 3).

## 4. Discussion

laBCC or mBCC have only restricted therapy options with HHIs as the most effective treatment to date. When first-line therapy with HHIs fails, consecutive PD1i is approved as second-line treatment. However, response rates for cemiplimab after HHI therapy are reported at only 24–31%, or stable disease control remains only temporary [6,7,8,9].

In this study, we show that a subgroup of patients can benefit from reinduction of HHI after pretreatment with HHI and PD1i. This sequential treatment was analyzed in 12 patients with laBCC or mBCC. We saw response or disease stabilization following initial HHI treatment in all patients. Whilst only one patient responded to the second-line PD1i, a mixed pattern of response was noted for HHI reinduction ranging from CR to PD as best response. Response rate to second-line PD1i treatment in our limited patient cohort was lower than previously reported (24–31% [6,7,8]). This might be due to a selection bias, since only patients with subsequent HHI reinduction were included.

Our results suggest that neither the response to initial HHI treatment, nor the reason for discontinuation of initial HHI, nor the type of aBCC (locally advanced or metastatic) predicted response to reinduction of HHI.

This is, to our knowledge, the first report on this clinically relevant scenario after approval of a PD1i for treatment of aBCC. There are only limited reports with sequenced therapy in aBCC at all.

Two French studies investigated efficacy of HHI rechallenge [10,11]. Both studies recorded CR in about a third of the respective patient cohorts with aBCC under initial HHI treatment with vismodegib, which was then discontinued. During follow-up, about 50% of these patients suffered a relapse of their aBCC after 18.4 or 24 months of treatment discontinuation. Patients with initial CR to HHI were rechallenged with a secondary HHI treatment cycle. Response to reinduction was seen in 65.7% and 85% of patients, respectively.

Comparison of our data to these two French studies is limited due to the fact that all our patients were reinduced with HHI irrespective of response to primary HHI. Nonetheless, response rates in our patient cohort following HHI reinduction were lower than in primary HHI, which is consistent with the observation of the two French case series. This confirms that reinduction of HHI can be a successful treatment option for a subgroup of patients with laBCC or mBCC. This is also in line with the MIKIE study, which reported that planned treatment interruptions (“drug holidays”) appeared not to impair efficacy of HHI [12,13].

However, the question occurs, as to why in some patients an initial acquired HHI resistance is reversible after treatment discontinuation due to progression and reinduction after intermittent PD1i, which was the case for three patients in our series. To explain this, different scenarios are possible.

First, epigenetic factors may play an important role in reversibility of resistance mechanisms. In this context, slow-cycling BCC cells have been identified, which are characterized by leucine-rich repeat-containing G-protein coupled receptor 5 (LGR5) expression and activation of the WNT signaling pathway. This slow-cycling state is reversible upon discontinuation of HHI treatment, leading to tumor relapse—yet is also re-inducible upon a subsequent cycle of HHI treatment, i.e., these tumor cells have been shown to respond again to HHI treatment [14,15,16]. A HHI treatment break or a sequential different treatment regime, such as PD1i, may even be of advantage to optimize secondary HHI response.

Second, mutations in SMO or in other HH pathway targets (upstream such as Protein patched homolog 1 (PTCH1), or downstream such as glioma-associated oncogenes 1/2 (GLI1/2) or v-myc myelocytomatosis viral-related oncogene (MYCN)) have been discovered to drive BCC progression and are also involved in acquired treatment resistance against HHI [17,18,19]. Different types of SMO mutations have been detected: either at the ligand/drug binding pocket or at distant sites [20]. Even though cross-resistance across different small-molecule SMO inhibitors were anticipated in these cases, clinical experience showed that switch of type of HHI can be beneficial for a subgroup of patients [21]. Among our three patients with response to HHI reinduction after eventual progression following initial HHI treatment, two received different types of HHI in the two treatment cycles.

With regard to AEs, our patients experienced similar AEs compared to previous studies. Our preliminary data suggest that there could be fewer adverse events at reinduction of HHI (Table 3). The mechanism behind that is unclear, and this finding needs to be fostered by a larger number of patients. This stands in contrast to the MIKIE study, where AEs were comparable irrespective of treatment interruptions [12,13].

Limitations of our study are the retrospective design and the small number of only 12 patients with diverse stages of aBCC, as some presented with mBCC and others with laBCC. Although our study suggests a benefit from HHI reinduction in a subgroup of patients, the results should therefore be considered as preliminary. Further data ideally from prospective studies are needed with more patients receiving an HHI–PD1i–HHI treatment sequence.

## 5. Conclusions

In summary, there is a potential of aBCC to respond to HHI re-exposure after HHI and PD1i treatment failure. Mechanisms leading to this reversible HHI resistance are unclear. Nonetheless, reinduction of HHI after interjacent PD1i can be a promising treatment option—with possibly less toxicity than during initial HHI treatment—for a subset of patients with laBCC or mBCC. This is especially relevant due to the currently still-limited treatment options in this scenario. To detect factors to support identification of this benefitting patient group, further analysis of potential biomarkers and of larger numbers of patients is necessary.

## Figures and Tables

**Table 1:** Patient characteristics.

Pat. ID	Sex	Age at HHI Initiation (Years)	Stage at Diagnosis	Site of Primary BCC or laBCC	Site of Metastasis at Diagnosis	Predisposing Disease
1	f	59	mBCC	Left flank	Lung, retroperitoneal	None
2	f	41	laBCC	Upper lip, inner corner of the left and right eye		Gorlin–Goltz syndrome
3	m	31	laBCC	Head		None
4	f	70	mBCC	Left mamma	Lung, bone, pleural, lymph node	None
5	m	77	mBCC	Right shoulder	lung, bone	None
6	m	87	laBCC	left ear		None
7	m	82	mBCC	Left flank	Subcutaneous, bone, lung	None
8	f	84	laBCC	Right ear conch		None
9	m	38	laBCC	Right shoulder		None
10	f	64	laBCC	Scalp	-	None
11	m	81	laBCC	Retroauricular right		None
12	f	66	laBCC	Corner of the left eye		None

HHI: hedgehog inhibition; BCC: basal cell carcinoma; mBCC: metastasized BCC; laBCC: locally advanced BCC.

**Table 2:** Course of treatment per patient, hierarchically ordered according to best response to (i) HHI reinduction, (ii) PD1i and (iii) primary HHI treatment.

Pat. ID	Type of 1st HHI	Duration 1st HHI (Months)	Best Response 1st HHI	Reason EOT 1st HHI	Type of PD1i	Duration PD1i (Months)	Best Response PD1i	Reason EOT PD1i	Time to HHI Reinduction (Months)	Type of 2nd HHI	Duration 2nd HHI (Months)	Best Response 2nd HHI	Reason EOT 2nd HHI	Further Therapy
10	Vismodegib	13.2	SD	Progress	Pembrolizumab	4	PD	Progress	18.2	Sonidegib	9	CR	Ongoing	-
1	Vismodegib	22.4	PR	Progress	Pembrolizumab	2.1	SD	No drug approval	27.6	Vismodegib	20.1	PR	Progress	Sonidegib/pembrolizumab
2	Vismodegib	64.5	SD	Progress	Pembrolizumab	6.5	SD	Pat’s request	6.5	Sonidegib	29	PR	Ongoing	-
8	Sonidegib	5.1	CR	AE	Cemiplimab	4.1	PD	Progress	27.4	Sonidegib	2	PR	Ongoing	-
6	Vismodegib/Sonidegib	22.3	PR	Progress	Cemiplimab	16.3	PR	Progress	18.3	Sonidegib	10.2	SD	Progress	-
9	Vismodegib	19.2	PR	Progress	Cemiplimab	8.1	SD	Progress	25.4	Sonidegib	13.2	SD	Progress	Cemiplimab
5	Vismodegib	26	SD	Progress	Pembrolizumab	5.9	SD	Progress	5.9	Vismodegib	4.1	SD	AE	-
3	Vismodegib	27	PR	Pat’s request	Cemiplimab	0.8	PD	AE	3.8	Sonidegib	3	SD	Pat’s request	Cemiplimab
4	Vismodegib	6.8	PR	Progress	Cemiplimab	2.1	PD	Progress	4	Sonidegib	3.1	SD	Progress	Carboplatin/paclitaxel/vismodegib/radiation
7	Vismodegib	25.4	PR	Progress	Cemiplimab	3.1	PD	Progress	4.1	Sonidegib	3	SD	Ongoing	-
11	Sonidegib	12.7	PR	AE	Cemiplimab	2.1	PD	Progress	20.4	Vismodegib	1	PD	Progress	-
12	Vismodegib	2	SD	AE	Pembrolizumab	5	PD	Progress	69	Sonidegib	3	PD	Ongoing	-

EOT: end of treatment; HHI: hedgehog inhibitor; PD1i: PD1 inhibitor; CR: complete response; PR: partial response; SD: stable disease; PD: progressive disease; AE: adverse event; pat: patient.

**Table 3:** Sequence of adverse events to therapy sequence; severeness grades (according to CTC-AE version 5.0) are given in brackets, if reported.

Pat. ID	AEs to 1st HHI	AEs to PD1i	AEs to 2nd HHI
1	Alopecia, weight loss, loss of appetite, muscle pain, arthralgia	None	Loss of appetite, weight loss
2	Alopecia, muscle spasm, depression	None	Muscle spasms, depression, weight gain, amenorrhea, brittle nails, fatigue
3	Alopecia, muscle spasms, weight loss	Vasovagal presyncope, myocarditis	None
4	none	None	None
5	Dysgeusia (I), alopecia (I), weight loss (III), muscle spasm (I)	None	Loss of appetite, weight loss (III)
6	Muscle spasms (I), dysgeusia (I)	None	None
7	dysgeusia, alopecia	None	None
8	Muscle spasm, arthralgia, dysgeusia, nausea, weight loss	Fatigue (I), weakness (I), pruritus (I)	None
9	Muscle spasms (II), dysgeusia (II), alopecia (III)	Exacerbation of psoriasis	None
10	Nausea, alopecia	None	Nausea
11	Muscle spasms (II), dysgeusia (III), weight loss (III)	None	Dysgeusia (II), weight loss (II)
12	Unknown	None	Myalgia, diarrhea, nausea

AE: adverse event; HHI: hedgehog inhibitor; PD1i: PD1 inhibitor.

## Data Availability

Data used in this study are depicted in the tables included in the manuscript.
